# Technological Development in Wholegrain Food Processing

**DOI:** 10.3390/foods14122009

**Published:** 2025-06-06

**Authors:** Francesca Nocente, Laura Gazza

**Affiliations:** CREA Research Centre for Engineering and Agro-Food Processing, Via Manziana 30, 00189 Rome, Italy; francesca.nocente@crea.gov.it

**Keywords:** whole grain, micronization, steam explosion, high hydrostatic pressure, extrusion cooking, ohmic heating, fermentation, sourdough, cereals, 3D printing

## Abstract

This review aims to give evidence of the current developments and potential applications of emerging technological methods to improve the technological performance and the sensorial acceptability of wholegrain products. The review explores the technologies based on physical, i.e., micronization, steam explosion, high hydrostatic pressure, extrusion cooking, ohmic heating, and 3D printing, and biotechnological methods, such as fermentation and enzymatic treatments in the pre-milling, milling, and transformation steps of wholegrain products. The literature from the past decade for this review article was collected from electronic databases such as ScienceDirect, PubMed, Google Scholar, and Web of Science.

## 1. Introduction

Currently, the consumer perception of food quality is associated with several attributes, which include appearance and taste, ease of preparation, least processability, nutritional and healthy value, affordability, and the safety and sustainability of the entire supply chain. Processing technologies represent a key factor in achieving these requirements, and cereal processing is one of the most important technologies in the food system since cereals represent a staple food for the entire world population. In addition to the presence of macronutrients, such as carbohydrates, proteins, and fats, cereals also supply important biologically active micronutrients, such as dietary fiber (arabinoxylans, beta-glucans, cellulose, lignin, and lignans), minerals, vitamins, and other bioactive compounds with antioxidant properties (sterols, tocopherols, tocotrienols, carotenoids, alkylresorcinols, flavonoids, tannins, and phenolic acids), which are proven to enhance health and wellness by reducing the risk of chronic diseases, such as cardiovascular diseases (CVDs), type 2 diabetes, colorectal cancer, and other types of cancers [1,2,3]. These health-related compounds are mostly located in the outer layers of the kernel; therefore, they are retained in wholegrain (WG) foods and removed in refined foods. Despite the superior nutritional and health value of wholegrain food, the presence of bran and germ leads to a decrease in both its shelf life and palatability, which represent the major constraints of WG processability and consumption. This, along with the need to stimulate the transition toward a circular economy model, promotes research and innovation in eco-friendly processing technologies that are capable of retaining nutritional and sensory qualities while also ensuring adequate shelf life. Finally, most of these emerging processing technologies are focused on the requirement of the industry for high-soluble dietary fiber foods due to the epidemiological evidence of the positive health benefits of soluble fiber in comparison to its insoluble form. Hence, since soluble dietary fiber is scarce in most fiber-rich foods, modifying the structure of dietary fiber to improve solubility using physical, chemical, and biological processes represents a promising strategy to increase soluble dietary fiber levels in a wider range of foods. The novelty of this review lies in its comprehensive approach to emerging food processing technologies applied specifically to whole grains, a focus that has been overlooked in the previous literature. While earlier reviews have predominantly examined individual processes, the present review integrates more technologies, either physical or bioprocessing methods, to provide a more holistic perspective. Moreover, previous works often reviewed findings across diverse food matrices. By concentrating solely on whole grains and systematically comparing multiple physical and bioprocessing techniques, this review fills a critical gap in the current body of knowledge and offers new insights into optimizing wholegrain processing for improved nutritional and functional outcomes.

## 2. Emerging Physical Technologies

### 2.1. Micronization

The presence of bran in wholewheat products represents the major hindrance to consumer acceptability because of its detrimental effects on technological properties and sensorial perception. Indeed, the inclusion of wheat bran, in addition to modifying the color, worsens the dough rheology and flavor of the end-products by affecting the loaf volume and crumb texture of bread and increasing the cooking loss of pasta, reducing overall sensory acceptability [4,5,6]. This is attributed to the insoluble dietary fiber in wheat bran, which can disrupt the gluten protein network structure, thereby reducing the dough’s processing properties [7]. Micronization, or ultrafine milling, is a mechanical size-reduction process that reduces particles to the micrometer scale. When applied to whole cereal grains, this technique finely grinds the entire kernel, allowing the preservation of bioactive compounds, such as dietary fibers and antioxidants. Micronization not only enhances the nutritional profile of wholegrain flours but also improves their functional properties, including solubility, bioavailability, and ease of incorporation into food products.

#### 2.1.1. Effect on Baking Performance

The reduction in bran particle size has been proven to improve the baking performance of wholewheat flour. A study by Li et al. [8] aimed to evaluate the influence of flour particle size on the baking properties of wholemeal flour using different wheat varieties with five different bran particle sizes. Their results show that medium and medium-large bran particle sizes produced bread with a higher loaf volume; on the contrary, the use of finer bran had a negative influence both on the hardness and chewiness of the bread, as well as causing it to be darker in color. Also, Alzuwaid et al. [9] found that milling coarse bran and the subsequent sieving of it to create smaller average particle size fractions had a negative impact on bread volume and texture. Although bread volume and texture are important quality parameters, they do not fully predict consumer preference, which may worsen with the addition of coarse bran compared to finer bran particles. However, to achieve an optimal balance between bread quality and phytochemical content, supplementation with coarse bran may be preferable. Moreover, some consumers might favor such bread if it is promoted as healthy and functional despite its potentially inferior sensory attributes [10]. Lin et al. [10] showed that the particle size of wholemeal flour plays a fundamental role in determining both the quality of bread and its digestibility. Three different particle sizes of wholemeal flour were examined, with an average size of 1315 (coarse), 450 (medium), and 199 (fine) μm. Dough made with wholemeal flour with a coarse fraction showed less extensibility and stability, and consequently, the bread had a more compact, less porous structure, smaller volume, and harder consistency, which are all considered poor quality attributes in bread. Furthermore, bread prepared with wholemeal flour with a fine grain size had a higher digestibility of starch than bread prepared with coarse and medium grain sizes.

#### 2.1.2. Effect on Phytochemical Extractability

In addition to improving the bread-making quality of wholewheat flour, small particle sizes increase the extractability of phytochemicals from bran. Alzuwaid et al. [9] reported that a reduction in the average size of bran particles influenced the composition of macronutrients and bioactive compounds; indeed, the finer fractions (<180 µm) had the highest protein and starch content compared to the coarser fractions. Moreover, the fine bran fraction had the highest phytosterol content, while the coarser fractions had the highest antioxidant content. Lai et al. [11] observed that micronized bran allows greater extractability of total proteins, phenols, and flavonoids. Furthermore, the fatty acid value (FAC), water-holding capacity (WHC), cation exchange capacity (CEC), cholesterol adsorption capacity (CAC), sodium nitrite adsorption capacity (SNAC), and in vitro antioxidant activity are increased when bran is micronized. Overall, the authors suggest that micronization can be used as a pretreatment method to improve the functional and antioxidant properties of wheat bran.

#### 2.1.3. Effect on Pasta and Noodle Quality

In addition to bran, micronization has been applied to the intact grain of durum wheat [5], einkorn wheat [12], and parboiled rice [13] to downsize the flour particles to the micron range (85% of particle <250 µm) to produce wholemeal dried pasta with sensory and cooking quality characteristics comparable to refined pasta. Lai et al. [11] explored the effect of reducing the particle size by ultrafine milling under different frequencies (0, 20, 30, 40, and 50 Hz) and constant time (30 min) of wheat bran on noodle quality. They found that wholewheat noodles supplemented with wheat bran particles as small as 26.50 μm, exhibited superior cooking, textural, and sensory properties compared to noodles containing either smaller or larger bran particles. Micronization has also been applied to Brewers Spent Grains (BSGs), again with the aim of reducing their grain size to improve their processability and, ultimately, their sensorial aspect. Nocente et al. [14,15] demonstrated that when micronized barley, einkorn, or tritordeum BSGs were added to semolina in a greater percentage compared to the BSGs, the pasta was enriched in bioactive compounds with a good compromise of processability and sensorial quality.

#### 2.1.4. Effect on Wheat Bran

Onipe et al. [16] studied the effect of dimensional reduction on the color and hydration capacity of wheat bran. The fine-grain size of the milled bran was indeed characterized by higher brightness and whiteness index values, whereas the medium-grain-size bran had the most intense color in terms of yellow. The authors suggest that the higher whiteness index of finer bran could make whole foods more attractive to consumers than coarse and medium-sized bran, and finally, finer bran can potentially be used in larger quantities to functionally enrich food products, such as bread, donuts, and biscuits, with less sensory implications. Lai et al. [17] explored the effect of micronization on the structural, functional, and antioxidant properties of wheat bran. They found an increase in protein, total phenols, total flavonoids, and fatty acid and antioxidant activity, indicating micronization could be used as a pretreatment method to improve the functional and antioxidant properties of wheat bran.

Micronization can also be combined with separation methods, such as size classification, air classification, or electrostatic separation, to separate kernel tissues efficiently and obtain flour fractions enriched with protein, fiber, alkylresorcinols, and arabinoxylans [18,19,20,21].

### 2.2. Steam Explosion

Another technological process that is able to reduce the particle size of wholemeal in order to improve its technological, sensorial, and nutritional quality is steam explosion (SE). This technology consists of high-pressure saturated steam pretreatment (170–210 °C) for a fixed time, followed by an instantaneous release of the pressure, which leads to a vapor expansion inside the fibers, causing a breakdown of the lignocellulosic matrix. Pang et al. [22] verified the effect of the pretreatment of wheat bran using SE compared to air-dried bran on milling efficiency, both in terms of the reduction in particle size and energy consumption at milling. The authors found that treating wheat bran using steam explosion before the milling process can effectively reduce energy consumption during the process itself, resulting in milled bran with a smaller particle size and excellent processability.

#### 2.2.1. Effect on Phytochemical Compounds

One of the main constraints in the use of bran as an ingredient is the presence of insoluble dietary fibers (cellulose, hemicellulose, and lignin) that affect the nutritional, technological, and sensorial features of wholegrain products [23]. Different pretreatments, such as mechanical, thermal, and enzymatic, have been applied to wheat bran to improve the bioaccessibility of health-promoting compounds to reduce antinutritional compounds, upgrade wheat bran storage stability by the inactivation of endogenous enzymes, and mainly increase the ratio of soluble fiber (inulin, pectin, β-glucan, galactomannans, mucilage, and soluble fiber dextrin) [24]. In particular, Aktas et al. [23] investigated the effect of SE followed by enzymatic treatment on the nutritional and technological qualities of bread integrated with SE-treated bran. They stated that SE treatment increased the solubility of the dietary fiber (DF) and decreased the phytic acid (PA) content of the bran-supplemented bread, and although the baking quality of the SE-treated bran was lower than that of the untreated bran, the enzymatic treatment largely compensated for the negative effect of the SE treatment, highlighting that SE has the potential to be used in food applications due to its nutritional benefits. Wang et al. [25] studied SE treatment as a method to improve the functional (water-holding capacity, swelling, and ion-exchange properties) and nutritional properties of wheat bran. The study highlighted that steam explosion can promote the conversion of insoluble dietary fiber into soluble dietary fiber and increase the content of water-extractable arabinoxylans, as well as break down the dense cell-wall structure of wheat bran to facilitate the dissolution of flavonoids and phenolic compounds, while the gluten was not weakened.

#### 2.2.2. Effect on Rheology and Shelf Life

Liu et al. [26] investigated the effects of incorporating treated wheat bran into wheat flour on textural and sensory properties. The addition of steam-exploded (SE) bran (0.8 MPa) decreased the peak viscosity, final viscosity, and setback while increasing the pasting temperature of the flour–bran mixtures. Furthermore, incorporating 6% SE wheat bran into chiffon cakes resulted in reduced hardness and chewiness, enhanced antioxidant activity, and an excellent overall quality score. In addition to improving technological and nutritional properties, steam explosion has been reported to be an economical and eco-friendly method for improving the shelf life of wholemeal products. To this end, Kong et al. [27] studied the effect of steam explosion on improving the preservation properties of wheat bran while maintaining or even improving nutritional properties. Indeed, compared to conventional thermal sterilization, steam-exploded wheat bran showed lower fatty acid and peroxide values, hindering the oxidation of lipids in wheat bran during processing and improving the potential use and preservation properties of wheat bran and reconstituted wholemeal flour. Furthermore, steam-exploded wheat bran showed higher soluble dietary fiber, total phenolic and flavonoid contents, and higher antioxidant activities. In addition, Kong et al. [28] investigated the effect of steam explosion on the bioaccessibility and physicochemical properties of wholemeal flour. They reported that SE treatment improved protein and starch digestibility and phenolic bioaccessibility in wholemeal flour. Some authors also reported that the chemical changes in wholemeal flour induced by steam explosion caused alterations in the flour’s solvent-retention capacity and rheological properties and altered the falling number.

#### 2.2.3. Application of SE on Other Cereals

In addition to whole wheat, the effect of the application of steam explosion has been investigated on other cereals. Bharathi and co-workers [29] applied steam explosion to the bran of perennial grain intermediate wheatgrass *Thinopyrum intermedium* (IWG) and observed complete deactivation of peroxidase and a significant increase in water-extractable arabinoxylans, free ferulic acid, and p-coumaric acid, accompanied by a significant reduction in phytic acid, even at the least severe condition, i.e., 130 °C for 5 min, even if the authors recommended severity factors of 2.5 and lower for IWG bran to prevent severe discoloration, minimize rancidity development, and obtain bran with suitable hydration properties for baking applications. Dang et al. [30] found that wholegrain barley treated with SE showed flour with good swelling power, water-holding capacity, and oil-holding properties, along with minimal bulk density. Similarly, Hong et al. [31] demonstrated that, under optimal conditions, i.e., a steam pressure of 2.34 MPa, processing time of 37.0 s, and initial moisture content of 10%, SE significantly enhanced the structure, hydration, and swelling capacity of *qingke* barley while preserving its nutritional value and antioxidant activity. Ziegler et al. [32] described SE as an effective pretreatment for producing fermentable sugars. Moreover, the storage stability of wheat bran is extended after steam explosion treatment due to enzyme inactivation [27,29].

The unique features of the SE process, such as a low dielectric constant of water, short residence time, and chemical-free extraction, make it particularly suitable for isolating high-value compounds, like phenolics and polysaccharides, from biomass. These applications merit further development as environmentally friendly alternatives to traditional solvent extraction methods; however, for heat-sensitive and hydrolyzable compounds, the high temperatures commonly used in SE require detailed optimization.

### 2.3. Extrusion Cooking

The organoleptic and nutritional properties of wholegrain foods can be modified by the extrusion-cooking process, along with decreasing the techno-functional problems related to their use. Indeed, it is a technological strategy intended for improving the chemical composition, expansion properties, pasting and hydration characteristics, texture, color, and functional components of wholegrain cereal foods [33,34], since it results in starch gelatinization, the cross-linking of proteins, endogenous enzyme inactivation, and the breakdown of antinutritional factors and causes mechanical damage to cell walls. Additionally, it promotes the development of flavor, increases the bioavailability of minerals and phenolic acids, and improves protein digestibility [35]. Furthermore, extrusion cooking has been shown to be an effective modification technique for wheat bran, reducing the recalcitrance of its structure, increasing short-chain fatty acid production upon fermentation, degrading phytate, and releasing ferulic acid [36]. Another significant benefit of the extrusion process is its ability to inactivate lipolytic enzymes, particularly in rice and corn bran, allowing for longer storage after inactivation. Desired product characteristics can be achieved by adjusting various extrusion parameters, including moisture content, barrel temperature, screw speed, pressure, and screw diameter. However, heat-labile vitamins may be destroyed by high temperatures during extrusion.

#### 2.3.1. Effect on Bran Microstructure

Cui et al. [37] reviewed the advancements in understanding the relationship between extrusion processing parameters, formulations, and the microstructure of wholegrain extrudates observed using scanning electron microscopy, micro-computed tomography (micro-CT), and confocal laser microscopy. They highlighted the complexity of regulating the microstructure of wholegrain extrudates because of the presence of many components, including starch, protein, dietary fiber, lipids, vitamins, and bioactive compounds.

#### 2.3.2. Development of Novel Products

The extrusion process can also mix multiple ingredients, which is helpful for the development of novel products [33]. Caporizzi et al. [38] investigated the development of innovative and nutritious gluten-free extruded breakfast cereals from blends of teff and rice flour, modulating the feed moisture and barrel temperature during the extrusion cooking process to meet the consumers’ acceptance. Oliveira et al. [39] studied the extrusion process using response surface methodology to investigate the effects of the replacement of wholegrain wheat flour by corn flour and extrusion conditions on the sectional expansion, density, texture, color, and water activity of expanded breakfast cereal development, including texture after soaking in milk. Roye et al. [35] reported that twin-screw extrusion cooking can modify the physicochemical and nutrition-related properties of oat bran. The inclusion of various types of extruded oat bran in food products can be tailored to specific goals or product requirements. For example, acid-extruded bran may be particularly suitable for products with an acidic flavor, such as fruit bars and yogurts.

### 2.4. High Hydrostatic Pressure

Thermal processing can often affect the nutritional and sensory quality of foods through the destruction of vitamins, modifications of texture and color, and the development of off-flavors. High-hydrostatic-pressure (HHP) technology is a mild non-thermal processing method that has gained prominence in the food industry for its ability to enhance the safety and shelf life of several foods by the inactivation of microorganisms and enzymes, preserving the nutritional and sensory properties of foods. This technology has the potential to address the needs of raising customer awareness, particularly of those who are looking for additive-free convenience or functional meals with better sensory and nutritional attributes [40,41]. In the HHP process, products are subjected to pressures of 100–1000 MPa using a fluid (usually water) to convey pressure simultaneously in every part of the treated food. The intense pressure primarily affects the non-covalent bonds of biomacromolecules, leading to protein denaturation, enzyme deactivation, and microorganism inactivation, hence providing a pasteurization effect [42].

#### 2.4.1. Effect on Nutritional Compounds

The degree of the changes in the physicochemical properties and functional activities of food macromolecules after HPP treatment depends on factors such as pressure levels, treatment time, and the specific characteristics of the food matrix [43], which determine the overall effectiveness and outcome of HPP. Conversely, low-molecular-weight molecules, including flavor substances, vitamins, and aromatic compounds, are generally less affected by HPP [44]. In recent years, HHP has also been used to enhance bioactive compound and nutrient contents, such as bioactive peptides, polysaccharides, polyphenols, GABA, slowly digestible starch and resistant starch, vitamins, and minerals and to reduce antinutritional factors and allergenic proteins [41].

#### 2.4.2. Effect on Rheology

Due to the characteristics of this technology to meet future requirements for minimally processed food, the use of HPP has also been extended to cereals, both in dough and baked food [45]. Indeed, HPP treatment induces starch gelatinization and the polymerization of proteins in cereal-based matrices, changing their functional properties and, consequently, the texture and structure of the products [46,47,48,49]. Regarding the effects of pressure on gluten proteins, it has been reported that pressures <200 MPa do not significantly affect these proteins because conformational changes occur only in the tertiary and quaternary structures of the proteins. On the contrary, pressure >200 Mpa affects gluten proteins by decreasing dough extensibility, increasing resistance to extension, and extending dough development times (*R*max) [50]. The other key factor responsible for the increase in the viscoelastic properties of HHP-treated wheat flour is starch because of the formation of hydrogen bonds between the partially gelatinized starch and gluten [50,51,52]. Indeed, HHP determines changes in starch properties, including water solubility, swelling power, pasting, water- and oil-holding capacity, thermal properties, and in vitro digestibility [53]. Shorstkii et al. [54] investigated the effects of three different high hydrostatic pressure (HHP) treatments (100, 300, and 600 MPa) on the water absorption, gelatinization properties, and microstructural changes of wheat grains. They found that HHP treatment accelerated the soaking process of the wheat grains, thereby positively influencing their pasting characteristics.

#### 2.4.3. Application of HHP for Gluten-Free Foods

Most of the research about the application of HPP technology is focused on gluten-free cereals to improve gluten-free (GF) dough quality due to the poor technological properties of these materials, i.e., the lack of protein network formation, poor gas retention properties, poor volume, and acceptability [49]. Cappa et al. [55] observed a significant increase in water-binding capacity, which improved dough consistency and processability, in rice flour treated with high hydrostatic pressure (HPP) at 600 MPa for 5 min at 40 °C. This treatment resulted in well-leavened bread with higher specific volume and improved crumb softness. Similarly, Seo et al. [56] investigated the impact of HHP treatment on the physicochemical properties of rice flour at different moisture contents. They found that water absorption capacity, solubility, and swelling power of the rice flour increased as both the moisture levels and pressure rose. Zhu and Li [57] studied the effect of HHP treatment up to 600 MPa on the physicochemical properties of quinoa flour and observed a decrease in viscosity during pasting, gel consistency, and in vitro starch digestibility, while the water solubility increased. They stated the HHP-induced changes in the physicochemical properties could be attributed to the gelatinization of the starch component and, to a smaller extent, to the changes in non-starch components.

### 2.5. Ohmic Heating

The basic principle of ohmic heating (OH) involves passing an electric current through raw food, causing instantaneous and homogeneous heating due to the conversion of electrical energy into thermal energy, also known as the Joule effect. OH generates heat directly inside the food matrix by passing an electrical current through it, with the amount of heat produced depending on the applied voltage gradient and the electrical conductivity of the food material. On the contrary, conventional cooking methods rely on traditional heating mechanisms, such as heat conduction and convection, which generally require extended cooking times. This leads to nutrient loss in wholemeal processed foods. Furthermore, ohmic cooking systems usually have a noticeable reduction in energy consumption compared to conventional rice cookers. However, ohmic heating faces constraints related to factors such as electrical conductivity, electrical field distribution, fouling deposits on electrodes, equipment design, and cost, although OH is also reported to show a significant reduction in energy consumption compared to conventional cereal grain cookers [58].

#### 2.5.1. Effect on Processability

A study measured the electrical conductivity of three varieties of brown rice and three types of whole grains (lotus seeds, red kidney beans, and Job’s tears) before cooking them using the OH method. When cooking these mixtures using conventional methods, lotus seeds, red kidney beans, and Job’s tears required precooking in boiling water before being combined with brown rice. In contrast, OH cooking eliminated this step, making it more convenient. OH also demonstrated better retention of antioxidant activity, as well as calcium, phosphorus, and potassium contents in the cooked samples compared to conventional methods [59]. However, OH may impact several functional characteristics, such as texture, color, the water absorption index (WAI), the water solubility index (WSI), and pasting properties. A study by Dias-Martins et al. [60] aimed to evaluate the effects of mechanical decortication on the physical properties of cooked pearl millet grains, as well as the optimal cooking time of decorticated and whole grains. The study also compared the quality attributes of pearl millet cooked using OH or conventional heating. Overall, OH did not affect the quality attributes of the grains and is considered an environmentally friendly and energy-efficient method.

#### 2.5.2. Effect on Nutritional Compounds

A recent review highlighted the impact of OH on the nutritional composition of food, focusing on microbiological safety aspects and preserving the nutritional quality of processed foods. However, commercialization of the OH technique is dependent on meeting food safety laws and guidelines since the recent literature reports that certain microbes may occasionally show signs of resistance to OH. There is a need to develop versatile, large-scale, and cost-effective ohmic heating equipment that incorporates an enhanced current control system to meet the demands of the food processing industry [61]. OH has also been proposed as a viable alternative for stabilizing wheat bran, which contains beneficial fibers, proteins, and minerals but is susceptible to deterioration due to high polyunsaturated fatty acids and enzymes. Stabilization through OH preserves these nutrients and prevents the formation of free fatty acids. Additionally, OH stabilization of wheat bran increases the vitamin E content and other nutritional compounds and improves taste, making it suitable for use in the food, fermentation, feed, and medicine industries [62]. In the context of corn processing, OH has been explored as an alternative to nixtamalization, a traditional maize preparation process in which dried kernels are cooked and steeped in an alkaline solution before being rinsed to remove the pericarp. The OH process has been reported to retain more phytochemicals and antioxidant capacity in corn meals than traditional methods, without the need for excess water or washing. This process preserves phenolic compounds, such as ferulic acid, in amounts comparable to wholegrain corn. Moreover, the OH technique offers an ecological advantage by reducing water usage and generating no effluents [63]. OH has also been applied to improve gluten-free (GF) bread properties, which are typically characterized by poor bread volume, color, and crumb texture since GF batters often have reduced viscosity, cohesiveness, and elasticity compared to wheat doughs. Research on OH in GF breadmaking indicates that it enhances bread quality by improving protein solubility, leading to OH-baked GF bread with better volume and overall quality than conventionally baked GF bread [64].

In summary, OH presents numerous advantages over conventional cooking methods, such as improved nutrient retention, energy efficiency, and minimal environmental impact. However, challenges remain in optimizing OH technology for large-scale use, addressing microbial resistance, and ensuring compliance with food safety standards.

### 2.6. Three-Dimensional (3D) Food Printing

Three-dimensional (3D) food printing is an emerging food processing technology that has the potential to revolutionize product development. Its ability to design customized, personalized food products creates the opportunity to produce visually attractive, sustainable, and multi-ingredient foods that also offer health benefits [65,66]. Indeed, this technology offers nutrition-specific solutions, addressing global health issues such as malnutrition, dysphagia, and obesity [67]. In addition to nutritional aspects, 3D food products can be customized in terms of color, shape, flavor, and texture, opening new possibilities for tailored food experiences [67]. Another benefit of 3D food printing is to broaden the range of food items by upcycling food waste or by-products into nutritious and attractive food products. This technology requires digitalization to create three-dimensional structures by layering food ‘ink’ ingredients in a sequential manner [68,69]. Currently, the application of 3D printing in the food sector mainly includes confectionery, dough, dairy products, fruits and vegetables, meat, and ingredients such as protein, starch, and hydrocolloids [70]. Different printing techniques have been adapted for the food sector, and extrusion-based 3D printing has found a broad range of applications, including the development of wholegrain-based food.

#### 2.6.1. Effect on Processability

Cereal-based materials (dough, paste, and batter) are particularly suitable for 3D printing due to their ability to maintain the 3D shape, but a deep understanding of the dough rheology in relation to printing behavior is necessary for the success of the entire printing process [71]. Guo et al. [72] used hot-extrusion 3D printing (HEP) for the first time to develop highland barley premade functional biscuits. They found that a 20% highland barley concentration with a 9% corn oil addition had better printability and moderate digestibility since the formation of the structures showed higher elasticity and stronger resistance to deformation. The biscuits had a lower predicted glycemic index and starch digestibility. One of the major constraints in developing suitable food ‘ink’ formulations for 3D printing is to guarantee the dimensional stability of the printed structures, in particular after post-processing, such as baking or boiling, which is required for making cereal-based foods palatable.

#### 2.6.2. Effect on Nutritional Compounds

In a study by Lille et al. [73], extrusion-based 3D printing was used to produce protein- and dietary fiber-rich snacks from whole milk powder and wholegrain rye flour. Trials with various ratios of milk powder and rye flour in the printing formulation revealed good printability and shape stability after printing in all formulations, whereas the shape retention during baking at 150 °C was improved by the presence of wholegrain rye flour, which imparted stability and rigidity during baking. A study by Habus et al. [74] evaluated the effect of bioprocessing wheat and amaranth bran with yeast, lactic acid bacteria, and inulinase, to remove fructans, as well as improve the quality of 3D-printed snacks. Bran bioprocessing has been shown to reduce the fructan content in both species, improve overall dough rheology, enhance the precision of 3D printing, minimize shrinkage during baking, and contribute to the desired texture of snacks. Combining 3D printing with bran bioprocessing enables the production of low-FODMAP snacks [74].

A comparison summarizing the advantages and disadvantages of each physical technology is reported in Table 1.

## 3. Bioprocessing

### 3.1. Germination

Germination involves the soaking of whole grains, followed by controlled sprouting. During this process, several enzymes such as amylase, protease, and lipases are activated, resulting in the breakdown of starch, protein, and lipids into simple sugars, free amino acids, peptides, free fatty acids, and other macromolecules, leading to enhanced nutritional value, including higher digestibility. Additionally, during germination, the *ex-novo* biosynthesis of several bioactive compounds such as tocopherols, flavonoids, tocotrienols, γ-aminobutyric acid (GABA) and phenolic compounds occurs [75,76,77,78], coupled with a reduction in antinutritional factors (e.g., phytic acid) and an improvement in the bioavailability of essential minerals, such as iron, zinc, and calcium [79]. Germinated whole grains can play a significant role in making wholegrain products more appealing and beneficial to consumers. As consumer demand for healthier and more natural foods increases, the use of germinated grains in food products presents an exciting avenue for the food industry.

#### 3.1.1. Effect on Nutritional Compounds

Kaur and Gill [80,81] observed that the germination of wheat, rice, oats, maize, barley, sorghum, and pearl millet caused a decrease in resistant starch due to its breakdown by amylolytic enzymes. Liu et al. [82] investigated the physiochemical properties of highly resistant starch rice during germination in order to apply high RS rice in the functional food industry. They concluded that germination treatment lowered the gelatinization temperature, making cooking easier while maintaining lower digestibility and a higher nutrient content. Moreover, they observed enhanced lipids, free glucose, polyphenolic compositions, flavonoids, GABA, and β-glucan contents. Also, Munarko et al. [83], studying different soaking conditions for brown rice germination, observed an increase in GABA, total phenolic and flavonoid content, and antioxidant activity. Li et al. [84] explored, in wholegrain sorghum, the effect of 48 h of germination on phenolic, flavonoid, anthocyanin, antioxidant capacities, inhibition capacity of α-amylase, bioaccessibility of phenolics, and digestibility of starch. Germinated sorghum seed results were higher in phenolics, flavonoids, and anthocyanins, as well as in their antioxidant capacity. Additionally, germination improved phenolic bioaccessibility by 10.18%, along with a reduction in starch digestibility by 13.87%. In the context of sorghum, Kayisoglu et al. [85] observed an increase in the protein and lipid content and a threefold increase in TPC in germinated seeds compared to ungerminated seeds of both red and white sorghum. Baranzelli et al. [86] observed that germination in soft- and hard-texture wheat cultivars remarkably increased the content of benzoxazinoids and the antioxidant capacity of the free phytochemical fraction, with the hard-texture cultivar displaying the greatest increase in antioxidant capacity upon germination. Aparicio-García et al. [87] used 96 h germinated seeds of oats to produce a novel gluten-free ingredient. Indeed, germinated oat flour results in an excellent source of protein (10.7%), β-glucan (2.1%), thiamine (687.1 μg/100 g), riboflavin (218.4 μg/100 g), and minerals (P, K, Mg, and Ca). Moreover, compared to ungerminated flour, it presented better amino acid and fatty acid compositions and a higher level of GABA, free phenolics, antioxidant capacity, enhanced protease and α-amylase, and reduced lipase activities.

#### 3.1.2. Effect on Baking Performances

The germination of wheat grain under controlled conditions could be used to increase wholegrain flour functionality in bread baking and consumer acceptability of wholegrain foods [88]. Cardone et al. [89] analyzed the effect of wheat germination on the bread-making performance of wholewheat flour and found improved bread height (~20%), specific volume (~15%), and crumb softness (~200%). Also, in the work of Nanumenko et al. [90], replacing 20% of refined flour with wholewheat flour derived from germinated grain led to improved farinograph quality index values. Indeed, the bread had a high specific volume and optimal rheological characteristics in terms of total deformation, plastic, and elastic characteristics. On the contrary, Stern et al. [91] observed that rheological characteristics were all negatively associated with germination duration and resulted in sourdough bread, which panelists perceived to have an inferior crumb. Moreno and colleagues [92] investigated the effects of two wheat grain germination periods (12 and 48 h) on dough extensibility and deformation resistance, as well as on bread texture, protein digestibility, and starch digestion rate. They observed that the germination of wheat grains significantly altered the chemical composition and functional properties of flour, increasing the lipid and protein contents after 48 h. Incorporating germinated flour into bread production improved the specific volume of the bread, though the texture did not surpass that of the bread made with non-germinated flour. Additionally, germinated flour enhanced the resistant starch and protein digestibility index. Dhillon et al. [93], in a study on the physicochemical and antioxidant properties of wheat-germinated flour, observed a higher protein content, oil absorption capacity, and water solubility index. The total antioxidant activity increased after germination, as well as the total phenolic content, which increased more than twofold. Interestingly, the overall acceptability score for all breads prepared with different ratios of germinated and ungerminated flour was high, particularly for bread prepared with 50% germinated flour, which received the highest score.

### 3.2. Sourdough Fermentation

Microbial fermentation is one of the oldest food processing methods used to produce cereal-based products, especially baked goods. It refers to the process by which microorganisms chemically modify food components through their metabolic activities, resulting in the biosynthesis of new compounds or the transformation of existing ones. In addition to the enhancement of nutrient bioavailability and functional properties, fermentation imparts novel aroma and texture, improving the taste, appearance, and safety of cereal-based products [94,95,96]. Traditional sourdough microbiota include lactic acid bacteria (LAB) and yeasts. *Lactobacillus sanfranciscensis*, *Lactobacillus plantarum*, and *Saccharomyces cerevisiae* are the predominant microorganisms involved in sourdough fermentation [95,97,98].

#### 3.2.1. Effect on Nutritional Compounds

Bioactive peptides, enzymes, organic acids, exopolysaccharides (EPSs), and vitamins produced by microbial populations are key metabolites responsible for the antioxidant, antimicrobial, and probiotic properties of fermented foods. These compounds play a significant role in the prevention of chronic diseases, such as obesity, diabetes, cardiovascular disease, cancer, and allergies [99,100].

#### 3.2.2. Effect on Baking Performances

According to the inoculum used, sourdough can be classified as type I, II, or III. Type I, used at the artisan level, exploits the microorganisms present in the dough, which results in spontaneous fermentation. In type II, a starter culture is added to sourdough fermentation, and it requires controlled parameters; hence, it is used in the baking industry. Type III combines type II with type I, and it is preferable for use in an industrial bakery due to the higher stability quality compared to fresh sourdough [101]. When sourdough is used, rather than *Saccharomyces cerevisiae*, for the bread dough fermentation, superior properties in the bread quality and technological value are achieved [102]. The longer shelf life of sourdough fermented foods is attributed to the inhibition of pathogenic microorganism contamination, a result of the antimicrobial activity of the fermenting microorganisms, which helps prevent food spoilage [103]. Moreover, it was demonstrated that sourdough fermentation reduces the predicted glycemic index (GI) in bread products. Demirkesen-Bicak et al. [104] observed a significant increase in resistant starch and, consequently, a decrease in the predicted GI in wholewheat sourdough breads obtained at a temperature of 30 °C using the type II fermentation method. This study suggested that the fermentation type and temperature could affect the GI and the textural and sensory properties of wholegrain sourdough bread. In addition, the sourdough fermentation of cereal bran is the most sustainable and promising option for improving the texture and taste attributes of wholegrain and fiber-rich products. Zhang et al. [105] studied the effect of solid-phase fermentation with different ratios of *S. cerevisiae* and *L. plantarum* on the physico-chemical properties of wheat bran and the quality of wholewheat bread. They reported an increase in the contents of polyphenols, soluble dietary fiber, and essential amino acids in wheat bran, and the quality of wholewheat bread in terms of a higher specific volume, more uniform porosity structure, better texture, and more volatile compounds was also observed. In their study, Wang et al. [106] proved that rice and wheat bran fermented with *L. plantarum* 423 had an enhanced odor intensity of both cereal brans. In addition, the fermentation broths showed good hydroxyl radical-scavenging activity and oxygen radical-scavenging activity, indicating fermentation is helpful in promoting the application of rice and wheat in healthy foods and nutraceuticals. Cera et al. [107] investigated the effects of sourdough obtained by different lactic acid bacteria and yeast starters consortia on the texture and flavor of 100% oat wholegrain bread. The sourdough oat breads were softer and had a higher specific volume and were rated with higher intensities of flavor and odor compared to the control bread.

#### 3.2.3. Effect on Adverse Food Compounds

Sourdough also reduces mycotoxin contamination in wheat-based products. Zadeike et al. [108] studied the effect of sourdough fermentation with *Lactobacillus plantarum* or *Pediococcus acidilactici* on the mycotoxin level contamination in wholewheat milling fractions. Prolonged sourdough fermentation (48 h) allowed a reduction in the DON content by 44–69% and removal of 15-acetyldeoxynivalenol (15-AcDON), alternariol (AOH), deoxynivalenol-3-glucoside (D3G), and toxins H-2 and HT-2, while the enniatin removal levels were in the range of 5–70%. Sourdough has also become the best-choice technological process to reduce FODMAPs in bread-making [109]. Longin et al. [110] compared the FODMAP content of different wheat flour varieties used in bread production under different sourdough fermentation timespans. They concluded that extended sourdough fermentation combined with low-fructan wheat varieties resulted in the lowest FODMAP content in bread. Similarly, another study demonstrated that wholegrain bread produced with sourdough exhibited a significant reduction in fructan levels, along with increased acidity, improved volume, and enhanced storage performance compared to bread fermented with baker’s yeast alone. Moreover, the use of specific starter cultures in sourdough fermentation reduced the fructan content by over 92%, yielding a low-FODMAP bread suitable for individuals with irritable bowel syndrome while also improving its nutritional and technological properties [111]. Torbica et al. [109] developed an industry-appropriate process for the production of innovative low-FODMAP breads from the wholegrain flour of wheat and rye with a high fiber content, thanks to the addition of fiber-enhancing additives (chia and psyllium). In another study using the application of biotechnological tools, including the use of endogenous (from wholegrain flour) and exogenous enzymes (from chicory root extracts, wheat malt, and baker’s yeast) and fermentation, coupled with process optimization, they obtained wholegrain low-FODMAP cookies and crackers based on wholegrain wheat and rye flours with high FODMAP contents and excellent sensory properties [112]. When applied together, these processes can have a synergistic effect on the nutritional quality of whole grains. In a study by Mencin et al. [113], the partial substitution of common white wheat flour for a bread recipe with various bioprocessed wholegrain spelt was investigated in order to improve the quality and sensory characteristics of the bread. Germinated seeds were subjected to fermentation with *Saccharomyces cerevisiae* or an enzymatic treatment with cellulase, xylanase, feruloyl esterase, protease, and α-amylase. The results indicate that incorporating 2.5% or 5% of ‘germinated + fermented’ spelt flour significantly enhanced the phenolic content and antioxidant activity of the bread, thereby improving its nutritional value while maintaining acceptable technological and sensory properties.

### 3.3. Enzymes

Enzymatic treatment has been mostly used to improve wheat bran functionality and nutritional profiles and represents a mild and eco-friendly technology suited to replace chemical additives, such as hydrocolloids and emulsifiers, hence contributing to the development of more clean and short labels.

#### 3.3.1. Effect on Nutritional Compounds

The enzyme treatment of wheat bran has a positive effect on a bulk of biochemical processes, such as the release of bound phenolic acids, solubilization of arabinoxylan, production of oligosaccharides, an increase in mineral bioaccessibility and the number of water-soluble antioxidants, soluble fiber, and free amino acids, improvement of technological properties, enhanced flavor profiles, and reduced starch digestibility and glycemic index [114]. In cereal grains, dietary fiber includes a soluble fraction (including arabinoxylans and other xylooligosaccharides, β-glucan, and fructooligosaccharides) and an insoluble fraction (including cellulose and resistant starch) [115]. The detrimental effects on dough rheological properties, as already mentioned, of the addition of dietary fiber are mostly due to the insoluble dietary fiber fraction. Indeed, a higher content of insoluble bran fractions yields higher water absorption, increased dough and bread firmness, crumb density, and, consequently, decreased loaf volume. This effect may be attributed to the high number of hydroxyl groups present in insoluble dietary fiber (IDF), which can form hydrogen bonds with both gluten and water, resulting in a denser texture and reduced loaf volume and height [115,116,117].

#### 3.3.2. Effect on Processability

In the production of wholewheat products, the most commonly used enzymes include the ones that hydrolyze non-starch polysaccharides, such as cellulase, xylanase, and β-glucanase. Microbial xylanases are being used in various industries, including food, feed, textile, and paper processing [118]. Xylanase converts water-unextractable xylan into water-extractable xylan, which improves bread characteristics in terms of reduced dough firmness and stickiness, increased bread volume, and a more uniform crumb structure [115]. In addition, the use of xylanase delays the staling of bread [119] and induces the release of xylo-oligosaccharides from flour, which exert pre-biotic effects, thereby promoting intestinal health [120]. He et al. [121] evaluated the combined effects of xylanase, lipase, and xanthan gum on the quality attributes and functional properties of wholewheat bread baked from frozen dough. Response surface methodology (RSM) allowed the selection of the optimal combination of enzymes to obtain bread from frozen dough with a higher specific volume, softer texture, better brown crumb color, and greater overall acceptability. Also, Sheikholeslami et al. [122] produced wholewheat bread using xylanase, alpha-amylase, and emulsifiers in different formulations. The use of xylanase alone or in combination with other enzymes showed higher water absorption as a consequence of the degradation of insoluble AX and better gluten network formation. Xue et al. [123] studied the effect of the enzymatic hydrolysis by β-endoxylanase and α-arabinofuranosidase on the nutritional and technological properties of wheat brans. The two-enzyme treatment was found to be more effective in reducing resistance to extension, softening degree, water absorption, and development time while improving the extensibility, stability time, porosity, and sensory characteristics of the steamed breads. Moreover, an increase in antioxidant capacity, soluble xylooligosaccharides, and phenolic acids was observed. Mohammadi and co-workers [119] investigated the effect of xylanase and pentosanase enzymes, alone or in combination, on the rheological properties of dough and baguette bread characteristics. They found that the addition of xylanase and/or pentosanase into the flour had no significant influence on the farinographic properties of the dough but improved its extensographic properties. Bread volume and crumb texture were improved by the use of enzymes, although the dosage of enzymes influenced the dough characteristics. On the contrary, no significant difference was observed in the physical shape, baking uniformity, taste, and odor of the bread crumb. A cellulase complex comprises three types of enzymes—endo- and exo-β-glucanase and β-glucosidase—which hydrolyze cellulose and cellobiase, respectively. The effect of cellulase treatment, as well as of other enzymes (α-amylase, glucose oxidase, maltogenic α-amylase, and xylanase), on the quality of wholewheat bread was studied by Tebben et al. [124]. They found that cellulase increased loaf volume and decreased crumb hardness and chewiness, whereas xylanase treatment improved bread volume, decreased crumb hardness, and slowed crumb staling. The effect of cellulase treatment in pasta and noodle preparation was also studied. Geng et al. [125], using ultrasound-assisted cellulase treatment on brown rice flour, obtained noodles with improved textural and cooking properties, along with lower starch digestibility and glycemic index. A lower predicted glycemic index was also observed in pasta obtained from durum semolina replaced by cellulase-treated wheat bran [126].

The main advantages and disadvantages of the bioprocess are highlighted in Table 2.

## 4. Driving the Future of Whole Cereal Processing

Emerging technologies in cereal processing are significantly transforming the production of functional foods by enabling the development of healthier, more sustainable, and consumer-oriented products. Innovative methods such as high hydrostatic pressure (HHP), extrusion cooking, ohmic heating, fermentation, sourdough techniques, and 3D printing are driving improvements in nutritional quality, microbial safety, and sensory characteristics while minimizing the need for additives and mitigating thermal degradation. HHP ensures non-thermal microbial inactivation with minimal nutrient loss, whereas extrusion and ohmic heating enable efficient processing, nutrient fortification, and the creation of novel textures. Fermentation and sourdough approaches enhance flavor, digestibility, and bioactive compound formation, supporting gut health and the development of functional profiles (Table 3). These advancements address longstanding challenges in conventional processing, such as nutrient depletion and limited valorization of cereal grains. The growing interest in natural, minimally processed, and functional foods has further accelerated the integration of these novel techniques, often in combination with traditional methods, to enhance nutritional value, reduce allergenicity, and promote food safety. Technological progress, including the adoption of diagnostic tools and digital manufacturing systems, enables real-time quality assessment, contaminant detection, and greater customization. In particular, 3D printing offers new possibilities for the on-demand production of nutrient-optimized, personalized foods aligned with individual health requirements. Moreover, the incorporation of alternative flours and functional ingredients contributes to extended shelf life and improved rheological and sensory performance. Industry trends such as mergers and acquisitions underscore the strategic importance of innovation, as companies seek to meet the increasing demand for organic, gluten-free, and high-protein cereals. As consumer awareness of diet–health relationships grows, cereal processing is poised to play a central role in next-generation food innovation, where sustainability, personalization, and technological integration converge to meet evolving market expectations.

## 5. Conclusions

The rapid advancements in wholegrain processing technologies are paving the way for improved nutritional quality, enhanced functionality, greater consumer acceptance, and sustainability of wholegrain-based products. Indeed, emerging technologies for cereal processing show great potential in modifying the structural and functional properties of wholegrains, improving digestibility while preserving essential nutrients, and enabling the production of innovative textures and novel formulations. In addition, these processes further contribute to enhancing the bioavailability of bioactive compounds and the ratio of water-soluble dietary fibers, reducing antinutritional factors, improving sensory attributes, and extending the shelf life of wholegrain foods.

Despite these technological advancements, challenges remain in scaling up these processes for industrial applications. Issues such as cost-effectiveness, regulatory approvals, and consumer perception must be addressed to facilitate widespread adoption. Future research should focus on optimizing these technologies to ensure sustainability while maintaining the nutritional integrity of whole grains. Interdisciplinary collaboration will be key to unlocking novel approaches that align with evolving dietary trends and environmental imperatives. By integrating these emerging methods, the food industry can enhance the appeal and accessibility of wholegrain-based products, ultimately contributing to better public health and more sustainable food systems globally.

## Figures and Tables

**Table 1:** Advantages and disadvantages of emerging physical technologies.

Process	Advantages	Disadvantages
Micronization	Moderate energy consumption and cost production; minimal denaturation of nutrients	Moderate initial cost investment for equipment
Steam Explosion	Low cost; simple and fast operation procedures; low chemical usage	High energy consumption; large initial cost investment for equipment; Maillard reaction; denaturation of nutrients; shortened shelf life; discontinuous production
Extrusion Cooking	Fast operation procedures; low chemical usage; versatility	High energy consumption and cost production; Maillard reaction; denaturation of nutrients; formation of harmful compounds and off-flavors
High Hydrostatic Pressure	Fast; low energy consumption; low chemical usage; minimal denaturation of nutrients; minimal formation of off-flavors; extended shelf life	Large initial cost investment for equipment
Ohmic Heating	Moderate energy consumption and cost production; fast; low chemical usage	Maillard reaction; moderate denaturation of nutrients
3D Printing	Minimal denaturation of nutrients; versatility and customizability	High energy consumption; shortened shelf life; large initial cost investment for equipment, software, and set up

**Table 2:** Advantages and disadvantages of bioprocessing.

Process	Advantages	Disadvantages
Germination	Increased content and bioavailability of nutrients; reduced content of antinutrients; low chemical usage	Time-consuming; continuous monitoring of the process; growth of harmful microorganisms
Sourdough Fermentation	Increased content and bioavailability of nutrients; low chemical usage; production of probiotics; extended shelf life	Time-consuming; continuous monitoring of the process
Enzyme treatments	Increased content and bioavailability of nutrients; low chemical usage	High cost production; continuous monitoring of the process; persistence of process residues

**Table 3:** The main beneficial effects of the innovative processing technologies on wholegrains discussed in the papers reported in the review.

Enhanced Nutritional Value and Biochemical Extraction	Enhanced Functional Properties, Texture, and Sensorial Perception	Increased Shelf Life	Reduced Energy Consumption
[9,10,11,23,24,25,26,28,29,31,32,34,35,37,38,41,53,59,63,65,66,72,74,75,76,77,78,79,80,81,82,83,84,85,86,87,93,96,99,100,104,105,109,110,111,112,113,114,115,120,121,123,126]	[4,5,6,7,8,9,10,11,12,13,14,15,16,17,18,19,20,21,23,25,26,29,30,31,33,34,35,36,39,44,45,46,48,49,50,51,52,53,54,55,56,57,60,64,71,72,73,74,82,88,89,90,91,92,93,94,95,96,101,102,105,106,107,115,119,122,123,124,125]	[27,28,29,37,42,61,62,103,108,111,119]	[22,37,58,60,63]

## Data Availability

No new data were created or analyzed in this study. Data sharing is not applicable to this article.
